# Concurrent outcomes from multiple approaches of epistasis analysis for human body mass index associated loci provide insights into obesity biology

**DOI:** 10.1038/s41598-022-11270-0

**Published:** 2022-05-04

**Authors:** Sheldon D’Silva, Shreya Chakraborty, Bratati Kahali

**Affiliations:** 1 Centre for Brain Research, Indian Institute of Science, Bangalore, 560012 India; 2 Interdisciplinary Mathematical Sciences, Indian Institute of Science, Bangalore, 560012 India

**Keywords:** Computational biology and bioinformatics, Genetics

## Abstract

Genome wide association studies (GWAS) have focused on elucidating the genetic architecture of complex traits by assessing single variant effects in additive genetic models, albeit explaining a fraction of the trait heritability. Epistasis has recently emerged as one of the intrinsic mechanisms that could explain part of this missing heritability. We conducted epistasis analysis for genome-wide body mass index (BMI) associated SNPs in Alzheimer’s Disease Neuroimaging Initiative (ADNI) and followed up top significant interacting SNPs for replication in the UK Biobank imputed genotype dataset. We report two pairwise epistatic interactions, between rs2177596 (*RHBDD1*) and rs17759796 (*MAPK1*), rs1121980 (*FTO*) and rs6567160 (*MC4R*), obtained from a consensus of nine different epistatic approaches. Gene interaction maps and tissue expression profiles constructed for these interacting loci highlights co-expression, co-localisation, physical interaction, genetic interaction, and shared pathways emphasising the neuronal influence in obesity and implicating concerted expression of associated genes in liver, pancreas, and adipose tissues insinuating to metabolic abnormalities characterized by obesity. Detecting epistasis could thus be a promising approach to understand the effect of simultaneously interacting multiple genetic loci in disease aetiology, beyond single locus effects.

## Introduction

Susceptibility to complex human diseases, example, type 2 diabetes and Alzheimer’s disease, among others are conferred by more than one gene as well as environmental influences. Technological advances in genomics have equipped researchers to make substantial progress in the field of genome wide association studies (GWAS) which is a robust hypothesis-free approach to scan entire genomes of individuals for identifying genetic loci that define susceptibility to complex diseases. GWAS have identified thousands of loci implicated in complex diseases unequivocally^1,2^. Nevertheless, complete understanding of the pathophysiology of complex diseases remains elusive through GWAS, and the genetic variations identified through GWAS explains only a fraction of the heritability of the disease^3^. GWAS carried out for adult human height, detected associations with common variants that could explain about 60% of population variation^3,4^. Near-independent genome-wide significant SNPs explain about ∼6.0% of the variance of BMI (by 785 SNPs) in ~ 700,000 European ancestry individuals^5^. However, by carrying out GWAS on a relatively small cohort size of 96 cases and 50 controls, a variant causing an amino-acid replacement in the complement factor H gene (*CFH*) was found to increase the risk of age-related macular degeneration (AMD) by sevenfold when occurring in homozygous state^6^. Also, just 52 genome wide associated variants explain more than half of the genomic heritability for AMD^7^. Thus, GWAS has been a huge or moderate success depending on the disease trait or phenotype of interest. It has also been proposed that undetermined heritability is due to incomplete linkage disequilibrium (LD) between low frequency or rare causal variants and genotyped SNPs, and thus not detected by stringent significance tests^8^. Another proposition is that rare variants contribute to the missing heritability and optimally designed rare variant association studies would highlight important biological pathways for understanding disease aetiology^9^. Furthermore, evaluating each SNP individually and enforcing stringent correction for multiple testing can result in crucial variants to be overlooked. This is a severe problem given that these variants arise from components of pathways and genes that work in tandem to impact the concerned phenotype^10^.

Additionally, Bateson proposed that a variant or allele at one locus could prevent a variant at another locus from manifesting its effect^11^. Bateson’s theory was corroborated by Sewall Wright^12^, where he acknowledged the relevance of gene–gene interaction by stating that selection pressure acts on the effects of the genetic background instead of individual genes. Shortly after, Sir Ronald Fisher described epistasis as the divergence from added effects on quantitative traits due to alleles at different loci^13^. Epistasis denotes the event in which the status of a trait arising from a genetic background deviates from the sum of its single locus effects, and can be interpreted as the effect of one locus being dependent on the allelic state of another or several other loci^14^. This idea has further been expounded when epistatic or gene–gene interactions among multiple loci in the genome have been observed for model organisms^15^. Even for human complex traits, it has been proposed that genetic interactions could explain some of the “missing heritability” undetected by GWAS^16,17^, and can thus act as a governing factor underlying the biology of non-Mendelian phenotypes^18^. Epistasis from a biological perspective represents the synergy of gene products that are part of interconnected pathways associated with the concerned phenotype^19^. Significant epistasis analysis in recent years have furthered our understanding of complex traits, such as, atrial fibrillation^20^, lung cancer^21^ and multiple sclerosis^22^, among others. In the pre-GWAS era, increased evidence for linkage at one locus had been observed when the interaction with another locus was considered for type 1^23^ and type 2 diabetes^24^ and inflammatory bowel disease^25,26^ showed that genetic variance for complex traits is predominantly additive and a large sample size of millions of unrelated individuals are needed for precise estimation of epistatic variance. Recently, successful epistasis in humans have been detected through genome screening projects^27,28^, and by statistical methods of epistasis analysis^29–31^. Campbell et al. has argued that studies about genotypic combinations in model organisms could facilitate our understanding for detection of epistasis in natural populations^32^.In this communication, we use the terms epistasis and genetic interactions interchangeably.

In this study, we have used population data from 785 individuals belonging to the Alzheimer’s Disease Neuroimaging (ADNI) cohort in order to detect second order genetic interactions for SNPs known to be associated with body mass index (BMI). A recent exhaustive review meticulously details the journey of identifying single locus effects associated with human BMI, and how multidisciplinary analytical approaches could enhance our understanding of the biology of obesity and identify newer treatments^33^. We employed nine different epistasis detection tools representing exhaustive, heuristics, and stochastic algorithms and identified 20 significant epistatic interactions for human BMI. We then take these top SNPs comprising the interactions for follow-up in the UKB imputed genotype dataset, constituting more than 188,000 individuals to carry out replication analysis. Our analysis shows that variants at genomic loci in or near *FTO* and *MC4R; RHBDD1* and *MAPK1*, exhibit reproducible pairwise interactions associated with human BMI.

## Results

### Statistical epistasis in human BMI-associated loci

We identified twenty significant pairwise interactions among BMI-associated loci (Supplementary Table S1), from the consensus results of nine tools in the ADNI dataset. We took forward the comprising SNPs in these interactions for replication in the independent imputed genotype dataset of UK BIOBANK for uncovering pairwise epistatic interactions associated with BMI.

We finally obtain two pairs of significant interactions in more than 188,000 individuals between rs2177596 (*RHBDD1*) and rs17759796 (*MAPK1*), rs1121980 (*FTO*) and rs6567160 (*MC4R*), from a consensus of nine different epistatic approaches. The detailed characterization of these interactions is given in the following sections.

### Pairwise interactions detected

One of the significant interacting SNP pairs in ADNI replicated in the UKB dataset, as a consensus obtained from SNPRuler and AntEpiSeeker- rs2177596 (A/T) and rs17759796 (C/A), (*p*_adj_ = 3.24E−02, 2.0E−02) (Table 1), with corresponding BMI increasing alleles T (β = 0.017, *p* = 5.61E−06), freq = 46% (ADNI), 42% (UKB) and A (β = 0.018, *p* = 4.2E−05), freq = 14% (ADNI), 15% (UKB) respectively. Additionally, one interaction, as a consensus of GMDR and MDR, between rs1121980(G/A) and rs6567160 (C/T) (*p*_adj_ = 0.001) was replicated independently in the UKB genotype matrix, with corresponding BMI increasing alleles: A (β = 0.079, *p* = 1.8E−142), freq = 44% (ADNI), 42% (UKB) and C (β = 0.056, *p* = 3.93E−53), freq = 23% (ADNI), 23% (UKB) (Table 1, Table S1). Moreover, rs7613875 (C/A) (*MON1A/RBM5*) and rs8050136 (C/A) (*FTO/RPGRIPL1*), identified as interacting in ADNI, failed to meet the criteria for permutation testing in MDR (cross validation consistency value of at least 7/10) in the UKB.

**Table 1:** Significant epistatic pairwise SNP interactions for BMI associated loci.

Interaction RSID		Coordinates				Interaction results from SNPRuler, AntEpiSeeker, MDR, GMDR	
		SNP1		SNP2		ADNI	UKB
SNP1	SNP2	Chromosome	Position	Chromosome	Position	*P*-adj**	*P*-adj
rs2177596 (T: 46%: 42%)	rs17759796 (A:14%: 15%)	chr2	227,890,283	chr22	22,190,163	0.0324, 0.02005 (SNPRuler, AntEpiSeeker)	0.0 (SNPRuler)*
rs1121980 (A: 44%:42%)	rs6567160 (C: 23%: 23%)	chr16	53,809,247	chr18	57,829,135	0.0392, 0.0229 (SNPRuler, AntEpiSeeker)	(GMDR) CVC : 8 Testing Balanced Accuracy (TBA): 0.5346 Cutoff TBA (*p* = 0.05): 0.5034 Cutoff TBA (*p* = 0.01): 0.5044 *p* value : < 0.001 (MDR) CVC: 9/10

Chromosome and position with respect to GRCh37.

*P < 10^−16^.

(Effect allele: Frequency in ADNI: Frequency in UKB).

**P-adj is based on 1000 permutation testing.

### Allelic combinations observed in individuals for significantly interacting SNPs

**Figure 1:**
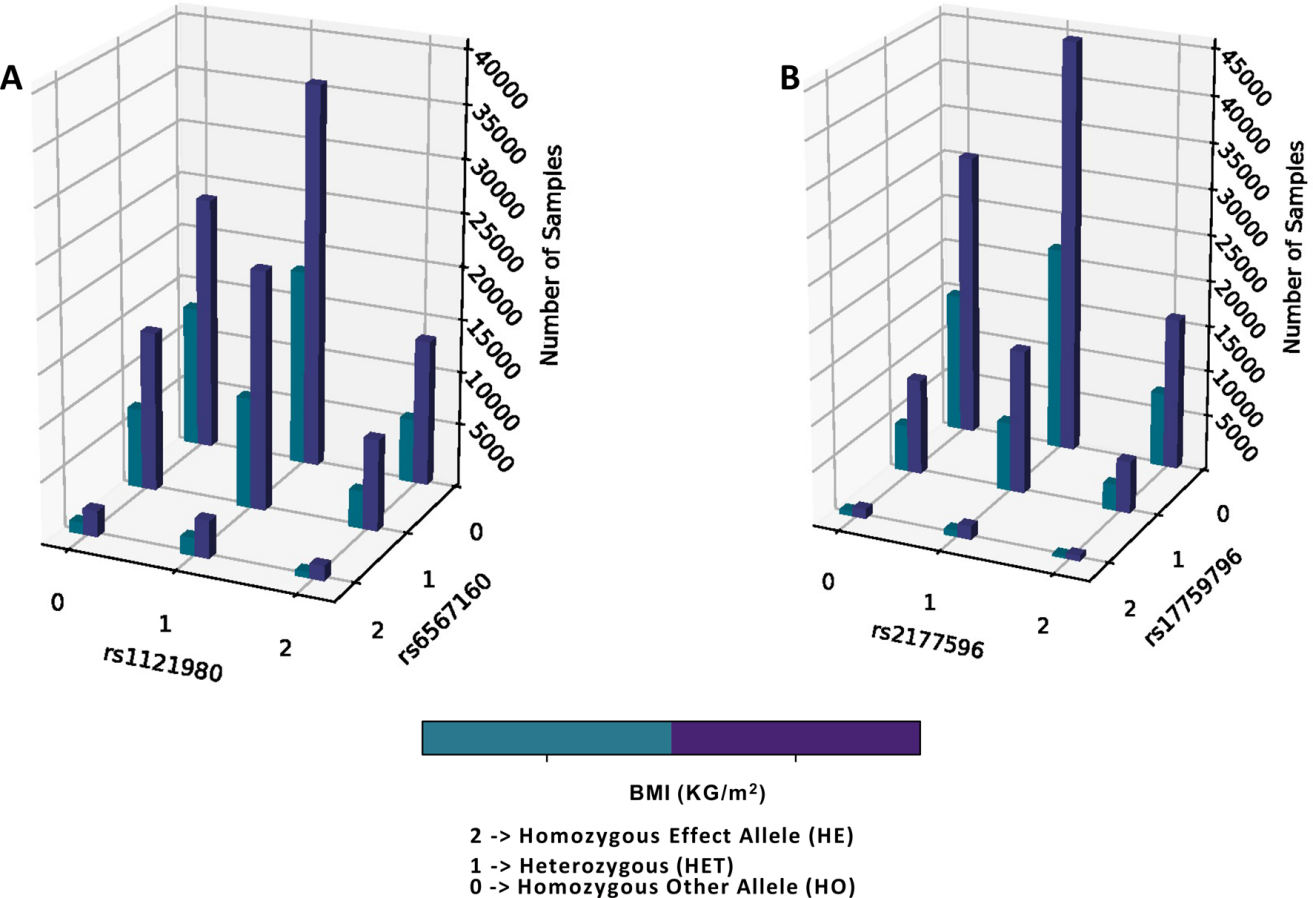
3-D plot showing three genotypes at each locus for the interacting pairs (**A** rs1121980 and rs6567160, **B** rs2177596 and rs17759796) with respect to BMI prevalence in UKB population. 3D barplot showing prevalence of obesity against genotypes at each interacting locus for two SNP pair interactions: (**A**) rs1121980 and rs6567160, and (**B**) rs2177596 and rs17759796.

We have investigated the genotype combinations of the two interacting SNPs against their prevalence in the UKB (Fig. 1). We observe that for every genotype combination in Fig. 1, the number of individuals is invariably more in the higher BMI group. This representation is based on absolute number of individuals carrying the said genotype combinations, and does not consider the total number of individuals in the high BMI and low BMI groups (126,651 and 61,969 respectively). There is marked difference in the total number of samples falling in the above-mentioned groups. When we closely observe the genotype combinations of effect and other alleles for the two interacting SNPs, individuals homozygous for BMI decreasing allele of both SNPs for the pair rs1121980 and rs6567160 were more likely to be predisposed to lower BMI as reflected in the UKB. This is because the proportion of individuals in the lower BMI group is significantly greater than the proportion in the higher BMI group; where proportions were assessed with respect to the total number of individuals in lower and higher BMI groups respectively (two sample z-test for proportions, *P* < 0.0001) (Table 2). Similarly, proportion of individuals heterozygous for rs1121980 and homozygous for the BMI-decreasing allele of rs65678160 are significantly more in the lower BMI group (*P* = 0.0025, Table 2). Proportion of carriers with heterozygous combination of rs1121980 and rs6567160 are observed to be predisposed to having increased BMI in UKB (*P* = 0.029, Table 2). In UKB, individuals who are homozygous for the BMI increasing allele for rs1121980, however, either heterozygous (*P* = 0.014) or homozygous carriers (*P* = 0.0185) of BMI decreasing allele for rs6567160, are more likely to exhibit higher BMI for this SNP pair (Table 2). None of the genotypic combinations for interacting SNP pair rs2177596 and rs17759796 show any significant over or under-representation of proportional carriers towards high or low BMI.

**Table 2:**
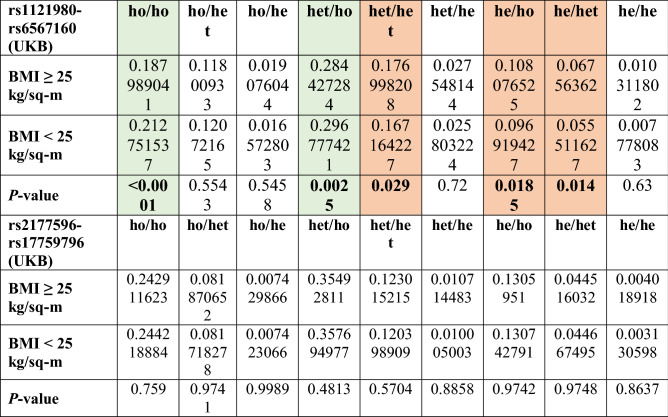
Combinations of allelic states for effect and other allele in individuals of high and low BMI.

Column headers he, ho and het represent homozygous for the BMI increasing (effect) allele, homozygous for the BMI decreasing (other) allele, and heterozygous states respectively.

Green cells represent the genotype combinations more likely to occur in low BMI rather than high BMI as per prevalence in UKB.

Orange cells represent the genotype combinations more likely to occur in high BMI rather than low BMI as per prevalence in UKB. Level of significance: 0.05

This analysis shows the importance of deciphering the simultaneous effects from multiple SNPs acting in conjunction to influence the phenotypes of our interest in large-scale population datasets.

Further, we examine the interaction effects versus main effects for rs1121980 and rs6567160 where BMI is regressed for the SNPs with age and gender as covariates (Fig. S1A), and we see that the proportion of variance explained by main effects is 0.0122 and that by including interaction term the proportion of variance explained becomes 0.0128. The interaction thus, explains 0.06% of the total phenotypic variance, which is not surprising given common variants like these can explain main-effects variance in the order of 0.008–0.3%^34^. Similarly, we examine the interaction effects versus main effects for rs2177596 and rs17759796 (Fig. S1B), where the proportion of variance explained by main effects is 0.00897 and that by including interaction term the proportion of variance explained becomes 0.00896. The interaction thus, explains 0.005% of the total phenotypic variance.

### Biological relevance of epistatic interactions

To systematically identify biological connections among the genetic loci uncovered for statistical epistatic interactions, we constructed interaction maps based on co-expression, co-localization, physical interaction, genetic interaction among the genes annotated to the interacting pair of SNPs.

**Figure 2:**
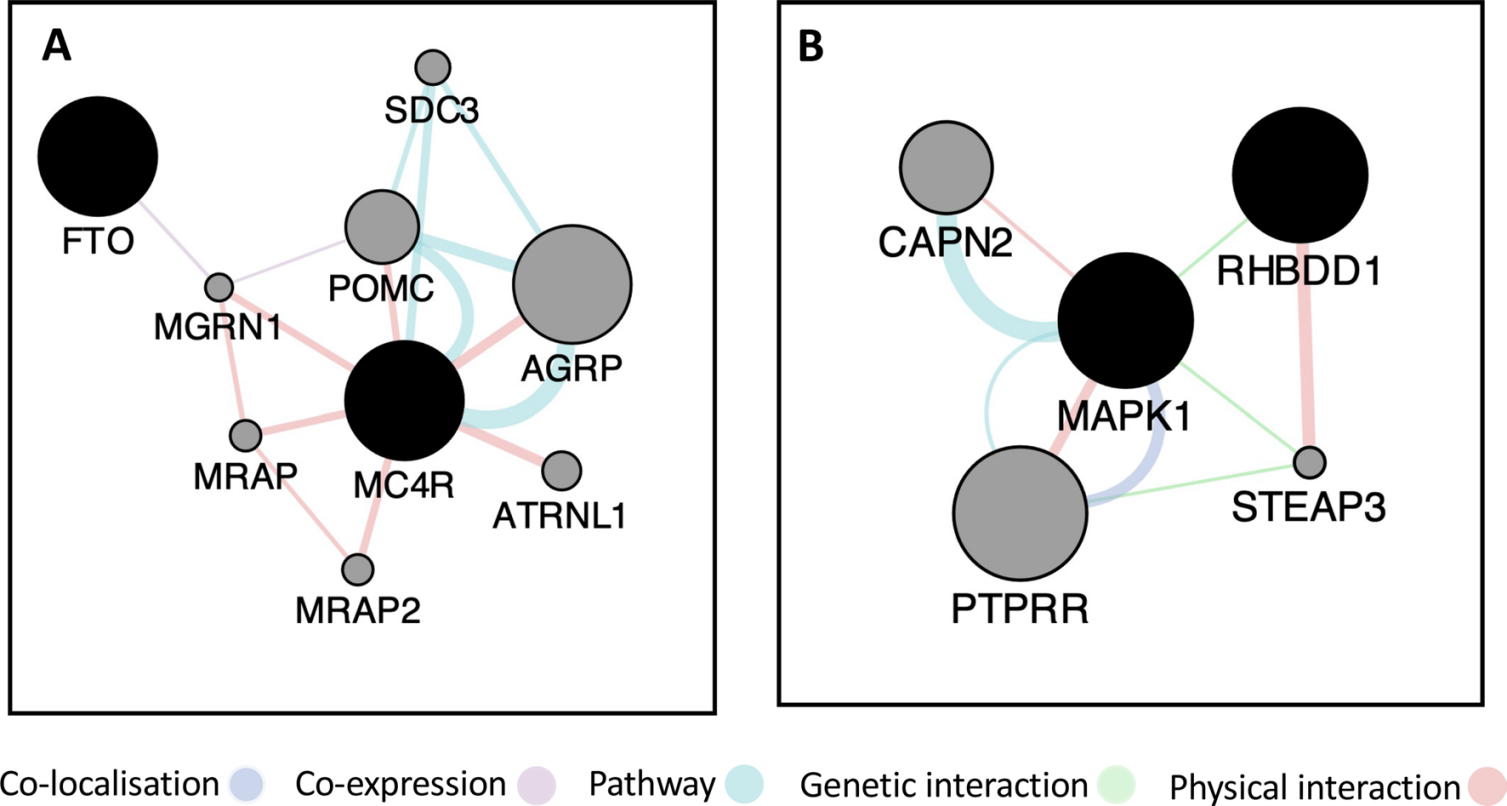
Gene networks. Nodes depicting genes are connected by edges formed on the basis of evidence for physical interaction (red), pathway (blue), co-expression (purple), co-localisation (violet) and genetic interaction (green). The maps for the detected interaction pairs are as follows: (**A**) rs1121980-rs6567160, (**B**) rs2177596-rs17759796. The solid black nodes are the query genes and the grey nodes are the resultant genes.

The gene network map of interacting pair rs1121980 (chr16:53809247) and rs6567160 (chr18:57829135) is annotated to *FTO* and *MC4R* respectively, as shown in Fig. 2A. Interaction between *FTO* and *MC4R* has been documented in only a couple of studies for obesity in children and adolescents^35^, and large artery atherosclerotic risk^36^, both studies done in less than 500 subjects, and for gene-diet interactions in 7052 individuals with high cardiovascular risk^37^.

Our results show for the first time that a pairwise interaction among SNPs mapping to *FTO* and *MC4R* is unequivocally associated with BMI in the UKB cohort of 188,620 individuals (after quality check). Our in-depth analysis reveals that *FTO* co-expresses with *MGRN1*^38^ which in turn shares a physical interaction with *MC4R*, and it has been shown that *MGRN1* inhibits *MC4R* signalling by displacement of Galpha(s), accounting for coat colour and obesity, features of the mahoganoid phenotype in mouse, and plays a key role in insulin sensitivity^39–42^. These results could further our understanding of the mechanism of energy intake and expenditure balance, with respect to satiety and weight loss or gain.

The gene network map for interacting pair rs2177596 (chr2:227890283) and rs17759796 (chr22:22190163) is shown in Fig. 2B. Genes annotated to rs2177596 are *RHBDD1* and *COL4A4*, the former is a functional component of endoplasmic reticulum-associated degradation for misfolded membrane proteins, and required for the degradation process of some specific misfolded endoplasmic reticulum (ER) luminal proteins, and apoptosis^43^, and is found to genetically interact with *MAPK1*^44^, a gene annotated to rs17759796. It is also known that many pathways in adipose tissue, liver, and pancreas can be disrupted during ER stress, and ER stress is one of the primary characteristics of obesity^45^. We thus report here the tissue specific expression results in liver and pancreas from GTEx for these two interacting loci.

### Expression profiles

**Figure 3:**
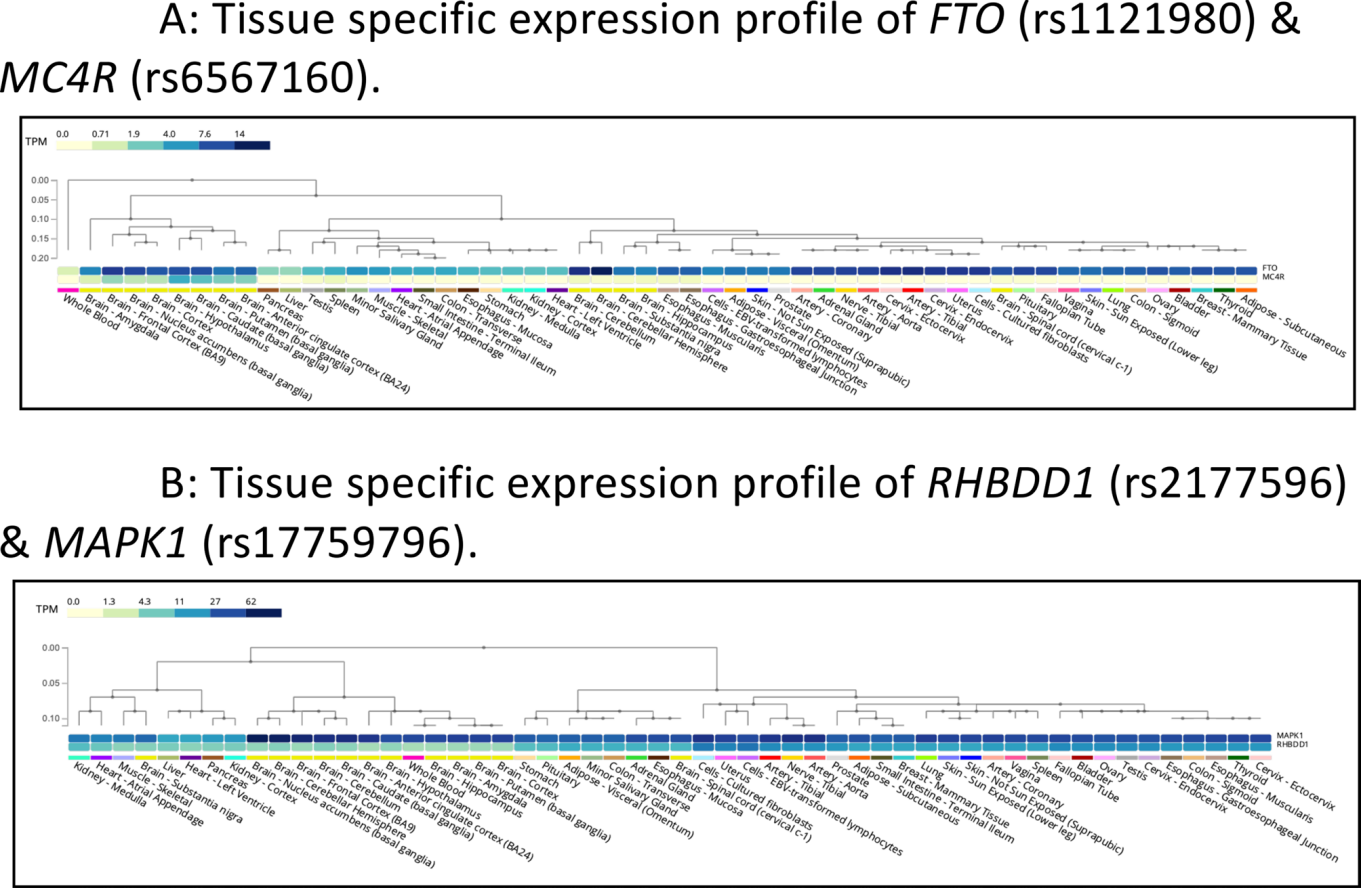
(**A**) Tissue specific expression profile of *FTO* (rs1121980) and *MC4R* (rs6567160). Comparison of expression (TPM) across various tissues, generated from GTeX for the genes *FTO* and *MC4R*. (**B**) Tissue specific expression profile of *RHBDD1* (rs2177596) and *MAPK1* (rs17759796). Comparison of expression (TPM) across various tissues, generated from GTeX for the genes *RHBDD1* and *MAPK1*.

**Figure 4:**
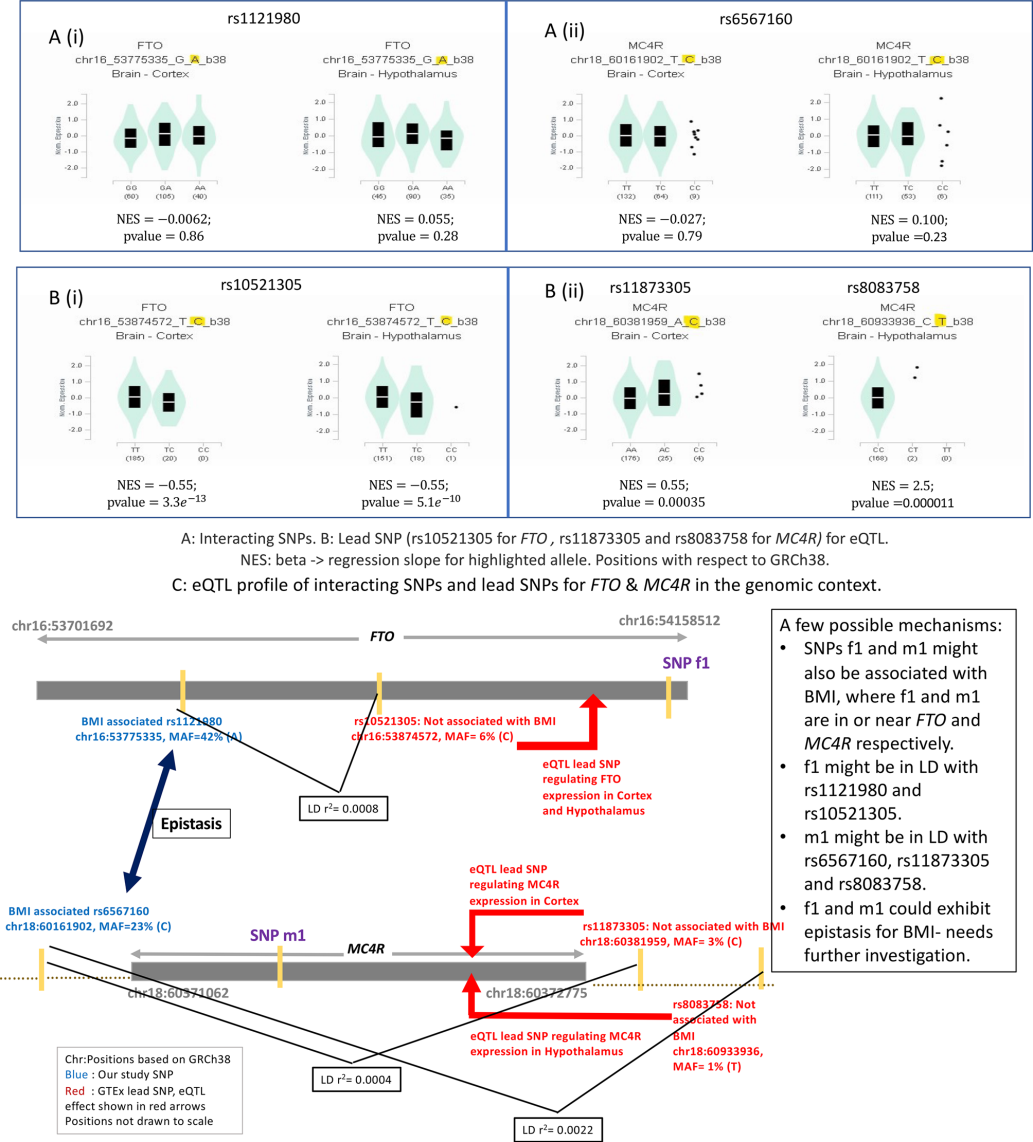
eQTL profile of FTO (i) and MC4R (ii) SNPs in relevant tissues. (**A**) eQTL profile of FTO (rs1121980) and MC4R (rs6567160) in brain cortex and hypothalamus. (**B**) eQTL profile of lead SNP of FTO (rs10521305) and lead SNPs of MC4R (rs11873305 and rs8083758 expressed in brain cortex and hypothalamus respectively). (**C**) eQTL profile of interacting SNPs and lead SNPs for FTO and MC4R in the genomic context. Possible mechanisms of action of epistatic SNPs in regulating expression of relevant genes.

Our results show a significant pairwise interaction between *FTO* (rs1121980) and *MC4R* (rs6567160) associated with human BMI in more than 188,000 individuals. Individually, both *FTO* and *MC4R* have emerged as strong signals for human obesity^34,46^. In mice, studies have shown that *FTO* can regulate fasting and feeding, and therefore variation in *FTO* can result in changes of feeding behaviour and obesity^47^. Recently, it has been shown that engineered deletion in the rs1421085 (r^2^ > 0.9 with rs1121980 in Europeans) conserved cis-regulatory module can affect organismal phenotypes relevant to obesity in mouse^48^. *MC4R* deficiency is known to be the most common cause of monogenic obesity^49^, and gain-of-function variants in *MC4R* is associated with lower risk of obesity, type 2 diabetes, and coronary artery disease^50^. We delve deeper into the individual-wise distribution of the *FTO* and *MC4R* alleles as explained in the previous section, and observe that carriers with rs6567160-TT (T is BMI decreasing allele) are predisposed to lower BMI in conjunction with rs1121980-AG, GG (N = 91,407). This is even more intriguing when we see that the entire UKB dataset has 61,969 individuals in the lower BMI category (*P* < 2.2 × 10^−16^ in a Binomial test). Thus, we posit that it might be possible that the BMI decreasing allele of rs6567160 near *MC4R*, contributing to its expression levels, has a significant mitigating effect on obesity in conjunction with the homozygous decreasing allele or heterozygous stature of the *FTO* SNP. *FTO* and *MC4R* are expressed in high levels in the hypothalamus and cortex in the brain as seen in GTEx tissue expression profiles (Fig. 3A). The eQTL analysis for BMI associated SNPs rs6567160 (BMI increasing allele = minor allele = C) and rs1121980 (BMI increasing allele = minor allele = A) in the hypothalamus and cortex is shown in Fig. 4A. They do not significantly regulate the expression of *MC4R* or *FTO*. Nevertheless, when we analyse the eQTL dataset for expressions of *FTO* and *MC4R* in cortex and hypothalamus, we notice that the lead SNP rs11873305 (Alt allele and minor allele = C) for *MC4R* significantly increases the expression (beta = 0.55, *P* = 3.5 × 10^−4^) of *MC4R* in cortex, while the lead SNP rs10521305 (Alt allele and minor allele = C) for *FTO* significantly decreases the expression (beta =  − 0.55, *P* = 3.3 × 10^−13^) of *FTO* in cortex. Similarly, significant increase and decrease of *MC4R* and *FTO* expression levels are observed in the hypothalamus for the corresponding lead SNPs rs8083758 (beta = 2.5, *P* = 1 × 10^−5^) and rs10521305 (beta =  − 0.55, *P* = 5.1 × 10^−10^) respectively (Fig. 4B). Thus, an increase in expression of *MC4R* in cortex and hypothalamus is accompanied by a decrease in expression to *FTO* in cortex and hypothalamus, with respect to the minor allele of these lead SNPs. However, these *FTO* and *MC4R* SNPs are low frequency (MAF = 1–6% in the European population) and not yet known to be associated with BMI, as well as not in LD (1000G European dataset) with the BMI-associated SNPs (rs6567160, rs1121980) for which significant epistasis has been identified in this study. rs6567160 and rs1121980 had been found to be associated with BMI from array-based genotypes and HapMap imputed studies. This makes us postulate that there could be another SNP in or near *MC4R* (say, m1) that is most likely to be causally associated with BMI, and in moderate LD with rs6567160 (BMI-associated), rs11873305 and rs8083758. Similarly, another SNP in or near *FTO* (say, f1) could possibly be the causal variant for BMI, and in moderate LD with eQTL lead SNPs rs10521305 and rs1121980 (BMI-associated). It would be motivating to uncover such SNPs f1 and m1 and investigate epistatic interaction between them, thus elucidating the network biology of obesity further (schema given in Fig. 4C). This observation from our results is especially interesting in the light of recent findings in an UKB-based study that gain-of-function variant [rs2229616 (coding change V103I), present in only 2% population] in *MC4R* can predispose individuals to lower obesity^50^, and we observe that rs2229616 is in LD with the cortex eQTL lead SNP rs11873305 (r^2^ = 0.22), which further provides evidence for the possible mechanisms we propose in Fig. 4C. With the technological advancements in genomics, when we uncover perfectly tagged variants by sequencing-based studies, discovery of epistatic effects would also be facilitated. Therefore, studying contribution from interaction effects of simultaneous genetic loci while considering the respective allelic combinations and uncovering eQTL SNPs in the same set of study individuals could facilitate our understanding of complex phenotypes.

rs17759796-A and rs2177596-T are respectively the BMI increasing alleles for *MAPK1* and *RHBDD1*. Our allelic combination analysis above do not reveal any statistically significant trend for belonging to higher or lower BMI for any of the nine possible genotype combinations for these two SNPs. We investigate the tissue-specific expression data for these two genes in GTEx. The two genes are expressed highly together in liver, pancreas, adipose-subcutaneous, and adipose-visceral tissues. In addition, *MAPK1* is widely expressed in several other tissues (Fig. 3B). Although the eQTL expression analysis of these genes in liver, pancreas, and adipose tissues (Fig. S2) could be indicative as to how they are modulated as a response to endoplasmic reticulum stress in obesity, the absence of a clear picture of any preferential allelic combination limits such in-depth investigation in this paper. There is also scarce genotype data for the lead SNPs for these two genes.

## Discussion

We present here a comprehensive perspective of how allele combinations from interacting SNPs can influence complex traits like human BMI. Our epistasis analysis shows that a diverse combination of trait increasing and trait decreasing allele with respect to their homozygous and/or heterozygous status for both SNPs in the interacting pair lead to differential susceptibility to obesity in the population scale, as the frequency of occurrence and the underlying biological function of the interacting SNPs play crucial roles beyond the additive mechanism of individuals SNPs in the manifestation of the trait in population scale.

We have detected and replicated two significant pairwise interactions for human BMI between genome-wide associated loci in more than 188,000 individuals. Interestingly, we uncover for the first-time interactions among SNPs near *FTO* and *MC4R* associated to BMI. Although, these genes have been implicated in BMI, and *MC4R* is known to play significant role in energy homeostasis, the role of *FTO* in regulating energy intake and expenditure is an active area of research. We show in a first, that they can be part of biological interactions via *MGRN1*, depending on the genotype combinations and allelic status of the interacting SNPs. This interaction result detected by GMDR in elucidating epistasis mechanism leads to new insights in governing human BMI in populations, that could be tested in functional studies. Additionally, independent SNPs rs7613875 (*MON1A/RBM5*) and rs8050136 (*FTO/RPGRIPL1*), identified as interacting in the discovery dataset, failed to meet the criteria for permutation testing in MDR (CVC value at least 7/10). Thus, future studies in *FTO* might shed more light for understanding the biology of obesity, and lead to our precise understanding of how *FTO* plays a role in energy intake and expenditure.

Our input list consisted of SNPs in established genome wide significant thresholds as well as sub genome wide significance levels, yet significant epistasis interactions have been uncovered for SNPs that were associated with BMI for single locus effects within *P* < 1E−04. This suggests that there are loci waiting to be discovered for governing the biology of obesity, and probing upto a threshold of comparable significance for SNPs already achieved in GWAS to uncover such latent interactions is useful.

This epistasis study on BMI implicated loci helped us identify mechanisms potentially implicated in the biology of BMI that cannot be captured by single locus effects analysis. Some of the networks we uncovered, especially the connections to energy homeostasis, endoplasmic reticulum stress, concerted expression profiles of interacting genes in liver, pancreas, adipose tissue, and hypothalamus have direct implications in obesity biology. In statistical epistasis, interactions are defined by genetic variations and not by physical interaction of biomolecules in real systems, the latter is also hard to establish experimentally. Even though statistical models can implicate genetic variants that do not occur in transcribed regions, for which the functionality in biological mechanisms becomes hard to experimentally prove, they are still our most desired approach, especially in humans, to provide insights into unknown biological mechanisms occurring through interactions among loci across the genome. Additionally, statistical models can define instances where multiple genetic factors have a non-additive effect on a phenotype. It has already been acknowledged^51^ that biological interpretation of epistasis is usually easiest when the penetrance values all equal either 0 or 1, leading to a clear relationship between genotype and phenotype. However, this explanation is not straightforward for human complex genetic diseases due to their polygenic and multifactorial nature. In humans, due to the complexity of genomic architecture and multitudes of factors influencing complex traits like BMI, our results are probably the tip of the iceberg and many more indiscernible genetic interactions among human BMI associated loci are waiting to be uncovered.

Despite their widely accepted potential role in capturing the phenotypic variance in traits owing to genetic effects, epistatic interaction in human genetic analysis has been debated in recent times. This is largely because of imperfect linkage disequilibrium between causal variants with large additive effect size and nearby tagging loci resulting in inflated test statistics for interaction terms. Wood et al^52^ reported that one more strongly associated variant uncovered from whole genome sequencing was moderately correlated with both of the interacting SNPs (showing very low levels of LD between them) identified by Hemani et al.^58^, and the inclusion of this third variant as a covariate removed any evidence for interaction. This means that the apparent epistasis reported by Hemani et al. for SNPs on the same chromosome can actually be attributed to a single causal allele having moderate levels of LD with each of the two SNPs, and has recently been dubbed as phantom epistasis^53^ between unlinked markers.

This is not the scenario in our results since we have detected inter-chromosomal interactions. On the contrary, our expression profile analysis reveals plausible mechanisms (Fig. 4C) involving causal variants at the epistatic loci that could be obtained by fine mapping.

Genetic variations making us susceptible to disease traits or determining the phenotypic variation in quantitative traits is a result of intricate selective forces at play striving to achieve the optimal balance in human systems. Epistasis is one of the intrinsic mechanisms on which such stabilising control ought to exist. Therefore, detecting statistical epistasis and deciphering biological implications for the same is of utmost importance in understanding disease mechanisms. Yet, we are cognizant that epistasis detection is limited by a number of factors. Statistical tests of interaction are limited to testing specific hypothesis in relation to genotypic measures in study subjects, and we will have insufficient statistical power to investigate all possible allelic combinations when dealing with rare variants. Additionally, when the causal variant or putative functional variant is in LD with another common variant, it is highly likely that the latter will show evidence for interaction in statistical tests due to linkage disequilibrium. In addition to these, inadequate controlling for overall genetic background, incidence of false positives, uncorrected environmental effects can constrain the identification of true epistatic effects present in human complex traits from statistical analysis.

Our results in discovery and replication dataset of more than 188,000 individuals highlight the importance of studying the interaction of SNPs for understanding the genetic architecture of complex traits. This is again more prominent in our in-depth analysis of the allelic combinations observed in individuals with high versus low BMI. We reiterate that our results are suggestively informing about plausible mechanisms to uncover putative interacting variants associated with obesity. We hope that our results will encourage large scale future studies to uncover epistasis in humans that will expound further the biology and genetic underpinnings of complex traits.

## Methods

**Figure 5:**
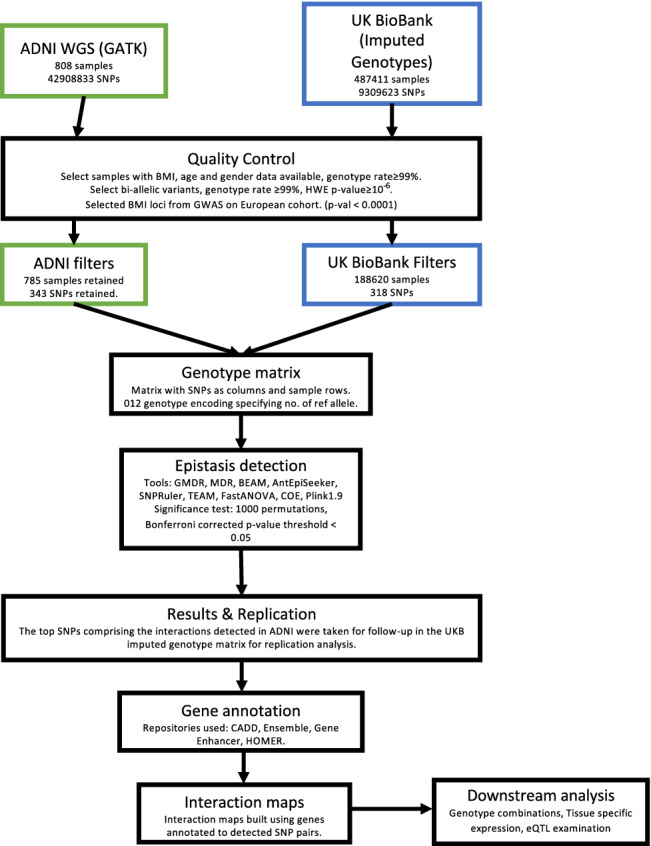
Study workflow. Schematic of procedures followed to detect and validate statistical epistatic interactions.

We considered BMI as our phenotype of interest in this work and set out to identify second order epistatic interactions among genetic loci associated with BMI at a genome wide level. The entire workflow is given in Fig. 5.

Data used in the preparation of this article were obtained from the Alzheimer’s Disease Neuroimaging Initiative (ADNI) database (adni.loni.usc.edu). The ADNI was launched in 2003 as a public–private partnership, led by Principal Investigator Michael W. Weiner, MD. The primary goal of ADNI has been to test whether serial magnetic resonance imaging (MRI), positron emission tomography (PET), other biological markers, and clinical and neuropsychological assessment can be combined to measure the progression of mild cognitive impairment (MCI) and early Alzheimer’s disease (AD).

### Creating a phenotype and covariate file

The Alzheimer’s Disease Neuroimaging Initiative (ADNI) along with UK BioBank (UKB) data repositories were used in this analysis, and we retrieved age, gender, height (in cm), and weight (in kg) for each participant. Among the 808 samples genotyped in the ADNI study, 785 had all the aforementioned fields. While 485,281 out of 487,405 samples with imputed genotypes in the UKB study had the required phenotype fields. UKB samples that self-identified as 'White British' and belonged to a similar genetic ancestry based on a principal components analysis of the genotypes (407,560 samples) were retained. From this set, 188,620 samples did not possess any missing genotypes, and were used for further analysis.

BMI for these individuals was calculated by formula (weight in kilograms)/(height in metres)^2^. Sample characteristics i.e., age and gender were parsed as covariates and BMI measure was selected as the phenotype. We have provided a threshold of BMI 25 kg/m^2^ in order to suffice the condition of certain programs used in our study that requires the user to specify a class variable for the phenotype, although the approach is linear regression to identify interaction scores, based on the guidelines laid down by the World Health Organisation with respect to the European population (https://www.euro.who.int/en/health-topics/disease-prevention/nutrition/a-healthy-lifestyle/body-mass-index-bmi).

In order to ensure that BMI is not confounded by cognitive status in the ADNI sample set, we compared the BMI distributions in the ADNI study cohort among samples in these three categories of cognition i.e., Dementia, Minimal Cognitive Impairment (MCI) and Control Normal (CN). We did not find any significant difference (assessed by *p*_adj_ < 0.01) in BMI between CN and MCI, CN and Dementia, MCI and Dementia or MCI + CN versus Dementia in Mann–Whitney–Wilcoxon test (Fig. S3).

### Selecting BMI Loci

We selected 466 genetic loci (Table S2) to be analysed for second order interactions for BMI by extracting SNPs associated with BMI from large scale meta-analysis studies^34,46,54,55^. We retrieved 359 loci, after extracting the genome wide significant and sub genome wide significant loci (*p*-value < 0.0001) reported in these publications and retaining the most significant SNPs among those present within 500 kb of each other.

### Generating genotype matrix

In order to rule out allelic bias due to any probable differential cognitive status among high and low BMI individuals, we checked for significant differences in allele frequencies of the 359 BMI-associated loci in a pairwise comparison of the CN, MCI, Dementia groups (Fisher’s exact test, and adjusted by Benjamini–Hochberg Procedure). One SNP in the *TOMM40* region (rs2075650) with significantly different frequencies (*p*_adj_ < 0.01) was removed.

For the ADNI genotype matrix, only bi-allelic variants without missing genotype values and Hardy–Weinberg equilibrium exact test *p*-value > 10^−6^ were retained. Out of the 466 loci (Table S2), 343 variants (Table S3) were selected for further analysis. And for the UKB genotype matrix, the variants with imputation r2 ≥ 0.7 were extracted for all chromosomes (39091537 variants). Additionally, biallelic markers with genotyping rate > 99% and Hardy–Weinberg equilibrium exact test *p*-value > 10^−6^ (35937211 variants) were further selected.

We generated the ADNI genotype matrix with 343 variants, where individuals were represented as rows and the variants as columns. Of the possible 398 loci obtained from the four meta-analysis studies on BMI (*p*-value <= 10^−4^), 318 were present in the UKB imputed genotype set (Table S3). The allelic states within each cell are represented as 0: homozygous reference, 1: heterozygous and 2: homozygous alternate allele.

### UKB replication set

Of the forty loci comprising the 20 interaction pairs obtained from the ADNI cohort, 8 loci (chr14:33302882, chr12:41948196, chr2:35404011, chr14:25928179, chr19:18454825, chr5:124330522, chr12:41921665, chr12:24075508) were not present in the UKB imputed genotype set, and the rest were investigated for interaction.

#### Epistasis detection tools

Multiple tools based on different classes of algorithms, for example, Exhaustive: FastANOVA^56^, COE^57^, TEAM^58^, MDR^59^, GMDR^60^, PLINK^61^; MCMC: BEAM^62^ and Heuristic: AntEpiSeeker^63^, SNPRuler^64^; have been developed and are generally used to statistically define gene–gene interactions or multi-order epistasis from large scale population data.

#### FastANOVA

FastANOVA^56^ relies on the application of ANOVA test for the detection of 2 locus interactions in a semi-exhaustive manner. It employs an upper bound which is the sum of one term derived from single-SNP ANOVA test, and the other derived from SNPs and independent of any phenotype permutation, to ensure effective SNP pair pruning.

#### COE

COE^57^ employs a similar approach like FastANOVA, where the single locus test and the genotype of the loci pairs are required to compute the upper bound and reduces the number of computations pertaining to permutation testing by grouping the SNP pairs according to their genotypes. The approach carried out by COE also makes for a tighter upper bound which results in an efficient SNP pruning mechanism and can be applied on all statistical tests that are convex by nature.

#### TEAM: tree-based epistasis association mapping

TEAM^58^ uses the construction of minimum spanning trees to define a contingency table that features the observed frequency of genotype permutations of each SNP pair related to its phenotype state. The table is then updated for every permutation of phenotype state across the population. Given that this method only considers those individuals with differing genotypes between SNP pairs, the computational burden to associate a SNP pair to the state of the phenotype is greatly reduced.

#### MDR: multifactor dimensionality reduction

MDR^59^ is a nonparametric method that uses machine learning to reduce a 3 × 3 dimensional genotype matrix into a binary classification of case and control. MDR trains its models on 9/10th of the input genotype matrix and carries out the testing phase on the final (10th) partition. Finally, the MDR model with the least prediction error and the most cross validation consistency is selected.

#### GMDR: generalized multifactor dimensionality reduction

GMDR^60^, based on the same principles as the MDR approach, can additionally handle quantitative phenotypes by taking into account a score statistic, generated by carrying out a generalized linear regression (GLM) with appropriate link function and suitable covariates, which is then used to map n-dimensional genotype combination into a 1-dimensional space. Final results are obtained after permutation testing.

*Plink 1.9*^61^.

#### Fast-epistasis

Calculates the odds ratio between loci A and B along with standard error. This procedure is performed for both cases and controls, and the epistasis test is defined as:

$$Z= (log(R) - log(S))/sqrt(SE(R)+SE(S))$$

where R and S are the odds ratios in cases and controls respectively.

#### Epistasis

Can input quantitative phenotype values and uses linear regression to fit the model

$$Y={\upbeta }_{0}+{\upbeta }_{1}{g}_{A}+{\upbeta }_{2}{g}_{B}+{\upbeta }_{3}{\mathrm{g}}_{A}{g}_{B}$$

for each inspected variant pair (A, B), where ${g}_{A}$ and ${g}_{B}$ are allele counts; then the ${\upbeta }_{3}$ coefficients are tested for significance.

PLINK does not support including covariates or carrying out permutation testing for epistasis tests.

#### BEAM: Bayesian epistasis association mapping

BEAM^62^ is a Bayesian implementation that defines both single locus and multi-locus disease association from case–control datasets. It classifies a pair of loci as interacting if their combined distribution better generalises the phenotype data compared to considering the loci independently. It uses Markov Chain Monte Carlo on a case–control SNP matrix to iteratively build the posterior probabilities of phenotype association for each marker.

#### AntEpiSeeker

AntEpiSeeker^63^ utilises Ant Colony Optimization (ACO) algorithm to identify statistical epistatic SNP pairs. Here, the SNPs being assessed form the probability distribution function that is updated through weights (pheromones). As the probability density function is fitted for the input genotype matrix, the epistatic interactions (paths) with higher corresponding scores get sampled by probes with increased frequency, resulting in these interactions being tagged with higher weight scores.

#### SNPRuler

SNPRuler^64^ employs predictive rule learning to identify and assess SNP interactions. With the premise that n loci interactions and phenotype class are governed by rules which can be found and evaluated qualitatively more efficiently than the interactions themselves. SNPRuler builds an enumeration tree with SNPs as nodes and phenotypes representing the leaves, it then prunes the search space using an upper bound on *χ*^2^ statistic.

All the tools described above and employed in this communication use permutation testing or multiple correction or in-built stringent *P* value threshold (*P* < 1E−4) to obtain robust epistasis results.

#### Hardware

We executed all the nine programs on a x86_64 architecture-based machine with an Intel(R) Xeon(R) Silver 4214 with 32 GB of memory (RAM) and CPU processing at a maximum clock speed of 3.2 GHz. The OS on the system was RHEL distribution CentOS 7.

#### Execution time

We directed COE, TEAM, FastANOVA, MDR and GMDR to carry out 1000 permutations. They clocked run times of 1 min 54 s, 16 s, 11 min 37 s, 16 min 8 s and 9 min 19 s respectively. BEAM, SNPRuler and AntEpiSeeker took 1 min 15 s, 1.14 s and 10 s respectively to output interactions at Bonferroni corrected *P* < 0.05. PLINK—fast-epistasis and—epistasis completed runs in 0.151 and 0.04 s respectively.

#### Memory utilisation

For executing the pairwise interaction analysis between 343 markers in 785 individuals, MDR had the highest memory consumption at 3200 MB. GMDR follows next with a consumption of 573.552 MB, followed by TEAM at 180 MB and SNPRuler at 180.06 MB. FastANOVA consumed 9.53 MB. BEAM, AntEpiSeeker had similar memory signatures with values of 2.84 MB, 2.18 MB respectively. COE consumed the least memory of 1.61 MB.

### Biological relevance of interacting SNP pairs

#### Generation of SNPs in linkage disequilibrium

We obtained the variants in high LD with the SNPs that emerged significant in our epistatic models, using the LDproxy tool^65^, from European sub-populations of 1000 Genomes Project (phase3). Only variants with R^2^ ≥ 0.9 were selected for further analysis.

#### Functional annotation of variants

We used each interacting SNP along with its corresponding LD variants as input for gene annotation in Combined Annotation Dependent Depletion (CADD)^66^, SNPNexus^67^, Hypergeometric Optimization of Motif EnRichment (HOMER)^68^ and gene enhancer regions^69^.

#### Mapping the genes onto networks

The gene annotations were then parsed to generate a gene interaction network for the detected epistatic models. The gene interaction networks were generated using GeneMANIA^70^, for the following subcategories- co-expression, co-localization, physical interaction, genetic interaction and shared pathways (Fig. 2).

#### Sample distribution by genotype states

For each of the detected SNP interactions, we extracted the number of samples for each genotype combination, thus resulting in nine combinations depending on the homozygous state for effect or other allele and heterozygous state of the two alleles for each pair of SNPs. We then compared the sample proportions in the high and low BMI groups for significant differences of these combinations using a two-sample z test for proportions (Table 2).

## Supplementary Information

Supplementary Information 1.

Supplementary Information 2.

Supplementary Information 3.

Supplementary Information 4.

Supplementary Information 5.

Supplementary Information 6.

Supplementary Information 7.

Supplementary Information 8.

## Data Availability

All phenotype and genotype data used in this study for analysis are available at ADNI (http://adni.loni.usc.edu/) and UK BIOBANK (https://www.ukbiobank.ac.uk). We shall share the in-house scripts as required by other researchers.
