# Supplementary material for: Concurrent outcomes from multiple approaches of epistasis analysis for human body mass index associated loci provide insights into obesity biology
Source: Sci Rep. 2022 May 4;12:7306. doi: 10.1038/s41598-022-11270-0 (PMC9068779; doi:10.1038/s41598-022-11270-0)
Supplement: Supplementary file 1 — Supplementary Information 1. [file 41598_2022_11270_MOESM1_ESM.pdf]

Figure S1: A) Main Effects vs Interaction Effects for SNPs rs1121980 (*FTO*) and rs6567160 (*MC4R*)

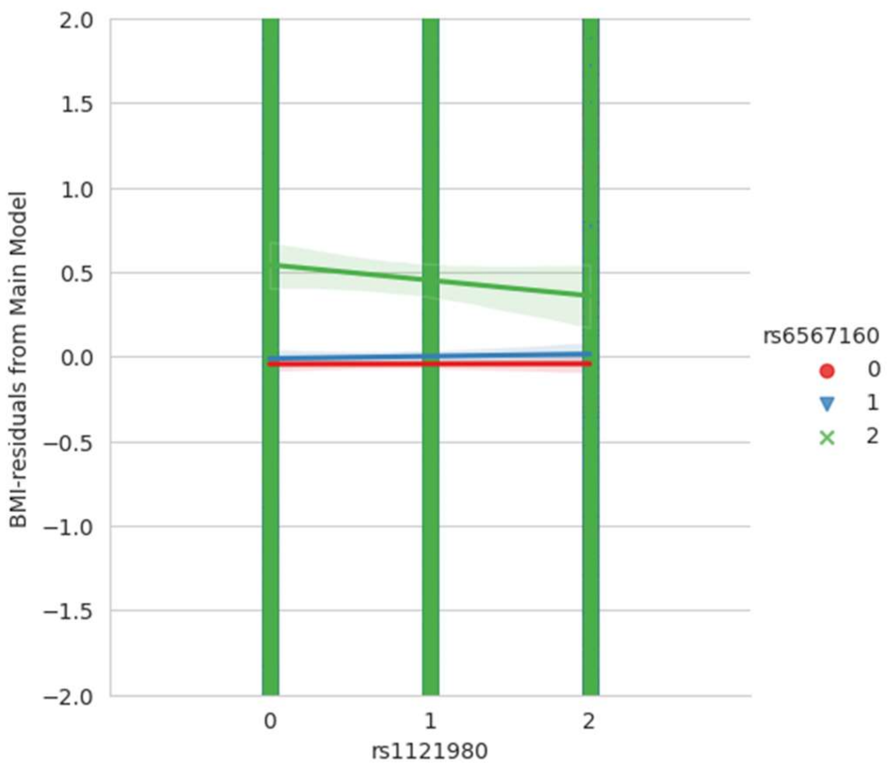

$$\widehat{BMI} = \mu + \beta_1 SNP_1 + \beta_2 SNP_2 + \beta_3 Age + \beta_4 Gender$$

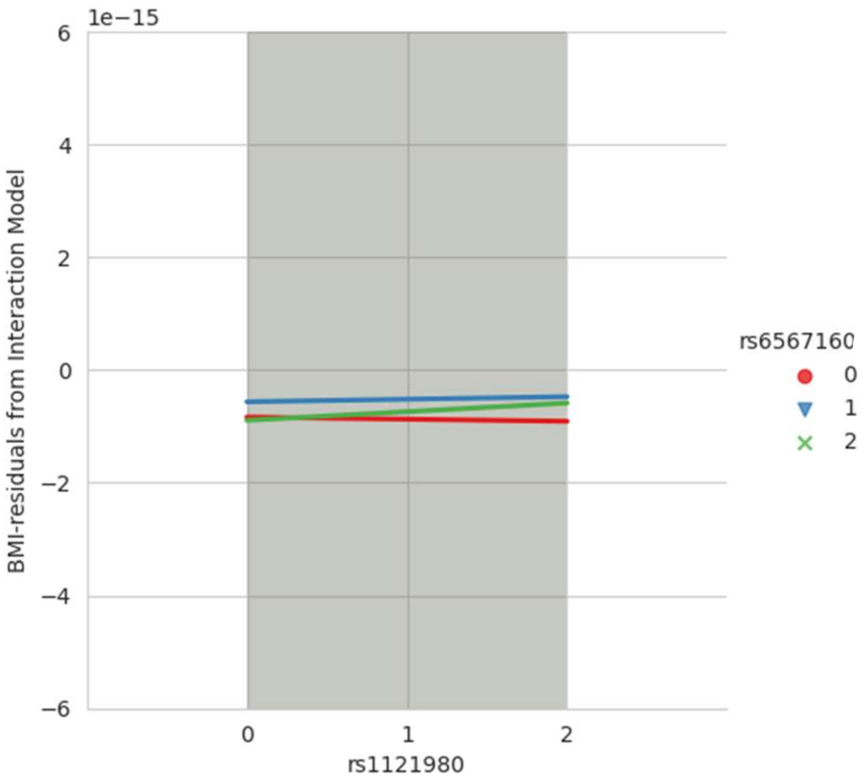

$$\widehat{BMI} = \mu + \beta_1 SNP_1 + \beta_2 SNP_2 + \gamma SNP_1 SNP_2 + \beta_3 Age + \beta_4 Gender$$
