# Supplementary figures and images for: Concurrent outcomes from multiple approaches of epistasis analysis for human body mass index associated loci provide insights into obesity biology
Source: Sci Rep. 2022 May 4;12:7306. doi: 10.1038/s41598-022-11270-0 (PMC9068779; doi:10.1038/s41598-022-11270-0)

Figure S2: eQTL profile of *RHBDD1* (rs2177596) & *MAPK1* (rs17759796) in relevant tissues.

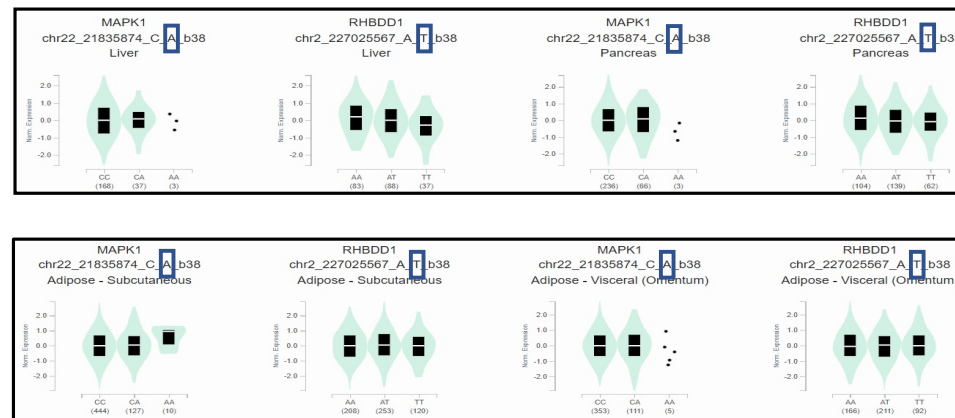

Supplement: Supplementary file 3 — Supplementary Information 3. [file 41598_2022_11270_MOESM3_ESM.pdf]

Figure S3: Comparison of BMI distributions in cognition groups.

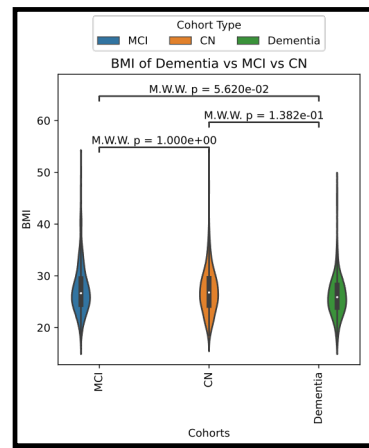

Supplement: Supplementary file 4 — Supplementary Information 4. [file 41598_2022_11270_MOESM4_ESM.pdf]
