# Supplementary material for: Concurrent outcomes from multiple approaches of epistasis analysis for human body mass index associated loci provide insights into obesity biology
Source: Sci Rep. 2022 May 4;12:7306. doi: 10.1038/s41598-022-11270-0 (PMC9068779; doi:10.1038/s41598-022-11270-0)
Supplement: Supplementary file 8 — Supplementary Information 8. [file 41598_2022_11270_MOESM8_ESM.docx]

LEGENDS TO SUPPLEMENTARY MATERIAL

Figure S1A

Title: Main Effects vs Interaction Effects for SNPs rs1121980 (*FTO*) and rs6567160 (*MC4R*)

Legend: Regression plot for the SNPs on BMI (main effects model-left panel) and additionally their interaction effect (interaction model in right-panel) with age and gender as covariates.

Figure S1B

Title: Main Effects vs Interaction Effects for SNPs rs2177596 (*RHBDD1*) and rs17759796 (*MAPK1*)

Legend: Regression plot for the SNPs on BMI (main effects model-left panel) and additionally their interaction effect (interaction model in right-panel) with age and gender as covariates.

Figure S2.

Title: eQTL profile of RHBDD1 (rs2177596) & MAPK1 (rs17759796) in relevant tissues.

Legend: eQTL profile of RHBDD1 & MAPK1 in liver, pancreas, adipose-subcutaneous, adipose-visceral tissues.

Figure S3.

Title: Comparison of BMI distributions in cognition groups.

Legend: Comparison of the BMI distributions of samples belonging to Dementia, MCI and CN diagnosis groups.

Supplementary Tables.

Supplementary Table 1: Significant pairwise epistatic SNP interactions for BMI associated loci from the ADNI genotype matrix.

Supplementary Table 2: Selected human BMI associated loci from published large-scale meta-analysis studies.

Supplementary Table 3: BMI loci selected after quality control steps.
